# *Ganoderma lucidum *polysaccharides can induce human monocytic leukemia cells into dendritic cells with immuno-stimulatory function

**DOI:** 10.1186/1756-8722-1-9

**Published:** 2008-07-21

**Authors:** Wing Keung Chan, Christopher Ching Hang Cheung, Helen Ka Wai Law, Yu Lung Lau, Godfrey Chi Fung Chan

**Affiliations:** 1 Department of Paediatrics & Adolescent Medicine, Li Ka Shing Faculty of Medicine, The University of Hong Kong, Hong Kong, PR China

## Abstract

**Background:**

Previous studies demonstrated *Ganoderma lucidum *polysaccharides (GL-PS), a form of bioactive β-glucan can stimulate the maturation of monocyte-derived dendritic cells (DC). The question of how leukemic cells especially in monocytic lineage respond to GL-PS stimuli remains unclear.

**Results:**

In this study, we used *in vitro* culture model with leukemic monocytic cell-lines THP-1 and U937 as monocytic effectors cells for proliferation responses and DCs induction. We treated the THP-1 and U937 cells with purified GL-PS (100 μg/mL) or GL-PS with GM-CSF/IL-4. GL-PS alone induced proliferative response on both THP-1 and U937 cells but only THP-1 transformed into typical DC morphology when stimulated with GL-PS plus GM-CSF/IL-4. The transformed THP-1 DCs had significant increase expression of HLA-DR, CD40, CD80 and CD86 though not as high as the extent of normal monocyte-derived DCs. They had similar antigen-uptake ability as the normal monocyte-derived DCs positive control. However, their potency in inducing allogeneic T cell proliferation was also less than that of normal monocyte-derived DCs.

**Conclusion:**

Our findings suggested that GL-PS could induce selected monocytic leukemic cell differentiation into DCs with immuno-stimulatory function. The possible clinical impact of using this commonly used medicinal mushroom in patients with monocytic leukemia (AML-M4 and M5) deserved further investigation.

## Background

In both Western and Oriental societies, cancer patients commonly take complementary and alternative medicine while they underwent conventional anti-cancer therapy [1-3]. Among different kinds of alternative medicine, herbal medicine is the most popular form taken by patients in United Kingdom [4]. In our community, more than 42% of our pediatric cancer patients took herbal medicine when they received conventional chemotherapy [5]. Among them, the commonest herb being used is the extracts derived from *Ganoderma lucidum*.

*Ganoderma lucidum *(GL) is a traditional Chinese medicine known by the layman as the "herb of immortality". It was used as a health tonic to promote longevity for more than two thousands years. It has two major groups of bioactive components: polysaccharides and triterpenes. In recent decade, they have been extensively studied because of its potential immunomodulating and anti-tumor effects as demonstrated in both *in vitro *and *in vivo *models [6]. So far, currently available data suggested that GL polysaccharides exert anti-cancer functions indirectly by activation of host's immune responses whereas GL triterpenes can kill cancer cells directly via its direct cytotoxic effect [7]. GL polysaccharides are purified from the mushroom mycelium and they contain branched β-glucan.

Dendritic cells (DCs) are the most potent antigen presenting cells and have unique ability in linking innate and adaptive immunity. Due to the scarcity of circulating DCs, the current protocol to study DCs biology and differentiation is mainly through differentiation of monocytes to DCs with the cytokines GM-CSF and IL-4. Recently, DCs can be induced from acute myeloid leukemic cells (AML) and this raised the possibility of using DCs derived from autologous leukemic cells for therapeutic uses [8]. Several AML cell-lines including monocytic THP-1, KG-1 and CD34^+ ^MUTZ3 cell-lines have been used as cellular models to study the differentiation of leukemic cells and DCs biology. However, the differentiation protocols differed greatly. For example, mature DCs could only be derived from THP-1 and KG-1 by adding GM-CSF and IL-4 together with ionomycin and TNF-α [8]. Interestingly, all study agreed that there is impaired response of leukemic DC to LPS directed DCs maturation [9]. This suggested that these leukemic DCs are somehow defective in response to maturation stimuli.

We and other groups demonstrated GL mycelium polysaccharides have the ability to stimulate the maturation of human DCs [10-12]. While most reports advocating the immunomodulating role of GL on normal monocytic cells, our data provided a novel observation that GL polysaccharides may also enhance monocytic leukemic cells proliferation and induce dendritic cells differentiation from monocytic leukemic blasts. The awareness of such phenomenon may help us to design specific treatment approach for monocytic leukemia.

## Results

### Cell proliferation response of THP-1 after GL polysaccharides stimulation

**Figure 1:**
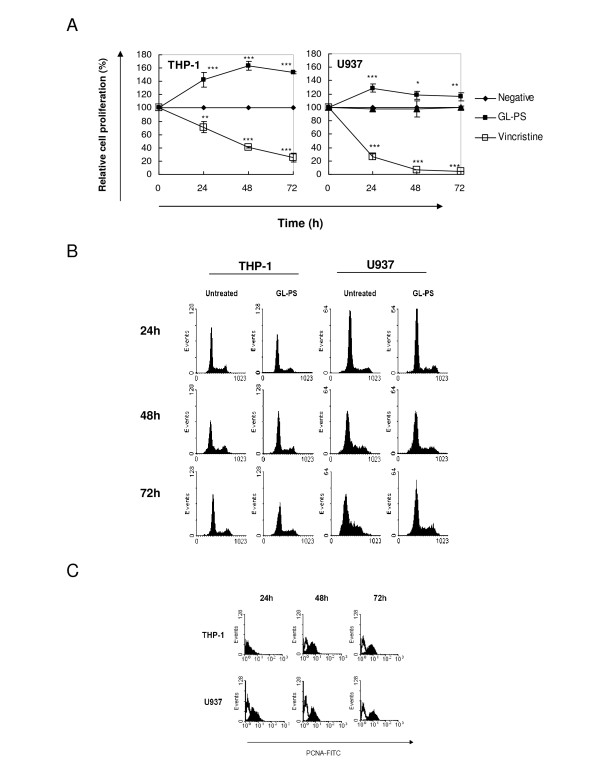
(**A**) **Leukemic cell proliferation induced by GL polysaccharides.** The time-response curves for the effect of GL polysaccharides (GL-PS, -▪-) at 100 μg/mL and vincristine (-▫-) at 0.1 μM on THP-1 (Left panel) and U937 cells (right panel) determined by XTT cell proliferation assay. The results represent the mean ± SD of triplicate cultures of three representative experiments. *p < 0.05; ** *p *< 0.01; ***p < 0.001 versus negative control (-•-). (**B**) Cell cycle analysis with PI staining. The THP-1 (left panel) and U937 cells (right panel) were treated with or without 100 μg/mL GL-PS for three days. The cell cycle analysis was then analyzed with PI staining and Cylchred Version 1.0.2 (Cardiff University, Wales, UK). The results shown were from one representative experiment of three independent experiments performed. (**C**) PCNA expression of GL-PS treated THP-1 (upper panel) and U937 cells (lower panel) in three-day incubation. The results shown were from one representative experiment of three independent experiments performed.

To relay the GL-PS has effect on leukemic cells, we evaluated the effect of GL polysaccharides (GL-PS) on the acute myeloid leukemia (AML) cell-lines THP-1 and U937 by cell proliferation assay. GL-PS alone at the dose of 100 μg/mL could stimulate the growth of both THP-1 and U937 cells. The average increases after the three-day exposure in THP-1 and U937 cells were 1.53-fold and 1.16-fold higher than the untreated control, respectively (Fig. 1A). Vincristine, a chemotherapeutic drug, was used as control to show the cells were responding. The cell cycle analysis with PI staining showed that GL-PS did not induce S phase arrest during the three-day treatments (Fig. 1B). By checking the expression of proliferating cell nuclear antigen (PCNA), which is an S-phase marker, both THP-1 and U937 cells showed increases in PCNA expression after GL-PS treatment (Fig. 1C).

### Induction of DCs-like morphology and phenotype in GL-PS treated THP-1 cells

**Figure 2:**
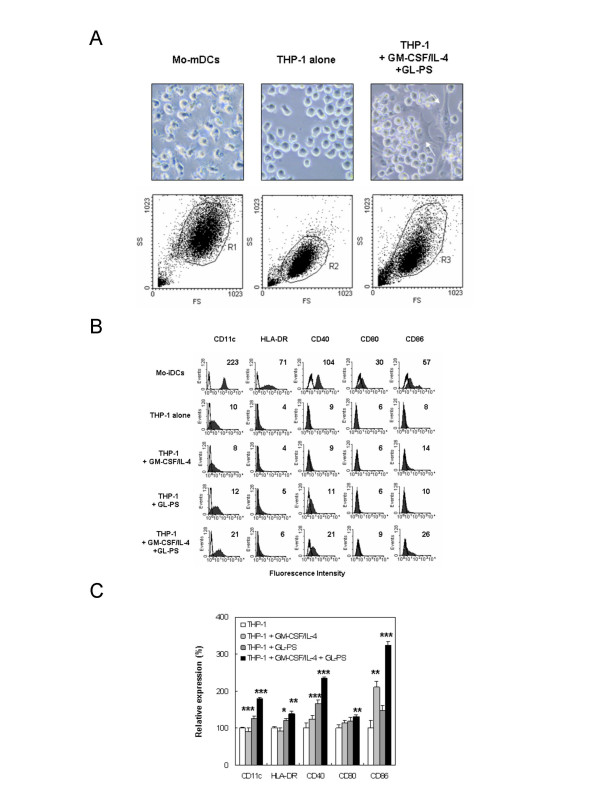
**Dendritic cell-like phenotype of THP-1 induced by GL-PS and GM-CSF/IL-4.** (**A**) Microscopic morphology of normal mature monocyte-derived DCs (Mo-mDCs) and THP-1 DCs (upper panel). The round THP-1 cells changed to be adherent flatten cells (white arrows). Under forward scatter and side scatter analysis of flow cytometer (lower panel), the THP-1 DCs increased in size when compared with THP-1 cells alone. (**B**) Surface expression of antigen presentation and costimulation molecules on immature Mo-DCs (Mo-iDCs), THP-1 cells alone, THP-1 stimulated with GM-CSF/IL-4; GL-PS treated THP-1, THP-1 stimulated with both GL-PS and GM-CSF/IL-4. The expressions of DC maturation markers CD11c, HLA-DR, CD40, CD80 and CD86 on DCs were analyzed by flow cytometer after five-day differentiation. The results shown were from one representative experiment of triplicate independent experiments performed. (**C**) The average relative expression of the DC maturation markers. The results were presented as mean ± SD of three representative experiments. *p < 0.05; ** *p *< 0.01; ***p < 0.001 versus that of THP-1 cells.

Under GL-PS treatment (100 μg/mL was used in all experiments onwards), we observed DC-like morphology in THP-1 cell culture. Since reports suggest that THP-1 can be induced into DC by a combination of cytokines [8,13], we hypothesized that GL-PS might also induce or enhance the differentiation of THP-1 cells into THP-1 DCs. We cultured these cells in the presence of GM-CSF/IL-4 with or without GL-PS. We used Mo-DCs as positive control and untreated THP-1 cells as negative control. THP-1 treated with GL-PS plus GM-CSF/IL-4 yielded atypical large adherent and elongated cells with multiple cytoplasmic spikes (white arrow) comparing to the round floating THP-1 cells and typical Mo-DCs with multiple satellite-like cytoplasmic protrusions (Fig. 2A). Under the forward and side scatter analysis of flow cytometry (lower panel), we found that the THP-1 DCs derived with GL-PS (GL-PS THP-1 DCs) had larger size as if the monocytes when differentiated into DCs. For U937 cells, we did not observe similar morphological changes (data not shown).

### Phenotypic maturation of THP-1 DCs derived with GL-PS

We then checked the surface expression of antigen presentation molecules and costimulation molecules, which Mo-DCs normally express. In Fig. 2B, we found that the THP-1 expressed relatively low levels of CD11c, HLA-DR, CD40, CD80 and CD86 when compared with normal Mo-DCs. Challenge of THP-1 with GM-CSF/IL-4 and GL-PS demonstrated increase in all markers when it was compared with the untreated THP-1 cells. To check the experimental consistency, we then normalized the fluorescence intensity from four experiments using the CD marker expression in THP-1 cells alone as the 100% (Fig. 2C). GL-PS alone could induce significant increase in CD11c, HLA-DR and CD40 when compared with the negative control THP-1 cells alone. But together with cytokine, the GL-PS THP-1 DCs showed significant increase in all five CD marker expressions, suggesting phenotypic maturity. The GM-CSF/IL-4 alone did not always increase the maturation marker expression in THP-1. To show the specificity of DC differentiation in THP-1 cells, we repeated the experiments on U937 cells. However, there was no significant increase in all DCs maturation markers (data not shown).

### Loss of cell proliferation response after added with cytokines

**Figure 3:**
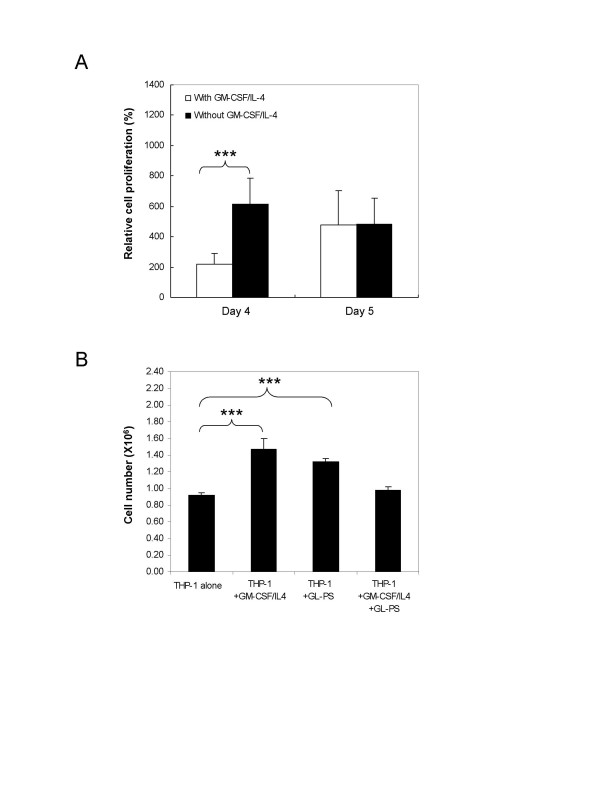
**Proliferation capacity of THP-1 cells treated with GL-PS after adding GM-CSF/IL-4.** (**A**) Effect of adding GM-CSF/IL-4 to THP-1 after three-day treatment of GL-PS as determined by XTT proliferation assay. The results represented the mean ± SD of three representative experiments. *p < 0.05; ** *p *< 0.01; ***p < 0.001 versus that without GM-CSF/IL-4 added. (**B**) Trypan blue exclusion assay. THP-1 cells after adding either GM-CSF/IL-4 or GL-PS were counted. The results represented the mean ± SD of three representative experiments. *p < 0.05; ** *p *< 0.01; ***p < 0.001 versus that of THP-1 cells.

To show the differentiation commitment of the GL-PS treated THP-1 cells in the presence of GM-CSF/IL-4, we added GM-CSF/IL-4 to the THP-1 cells, which had been treated with GL-PS for three days. We monitored the cell growth for two more days (Day 4 and 5) by XTT proliferation assays (Fig. 3A). Adding GM-CSF/IL-4 induced significant increase in proliferation in GL-PS treated THP-1 on Day 4. However, the proliferation became static after Day 5. We confirmed the cell proliferation results with cell counting using trypan blue exclusion assay. As shown in Fig. 3B, we recorded significant increase in cell number when either GM-CSF/IL-4 or GL-PS was added to the THP-1 cells on Day 5. When both GL-PS and GM-CSF/IL-4 were added, the cell number retained similar to the negative untreated THP-1. The decrease was not due to the cell death as indicated by the trypan blue staining (data not shown).

### Upregulated endocytotic activity of THP-1 DCs derived with GL-PS

**Figure 4:**
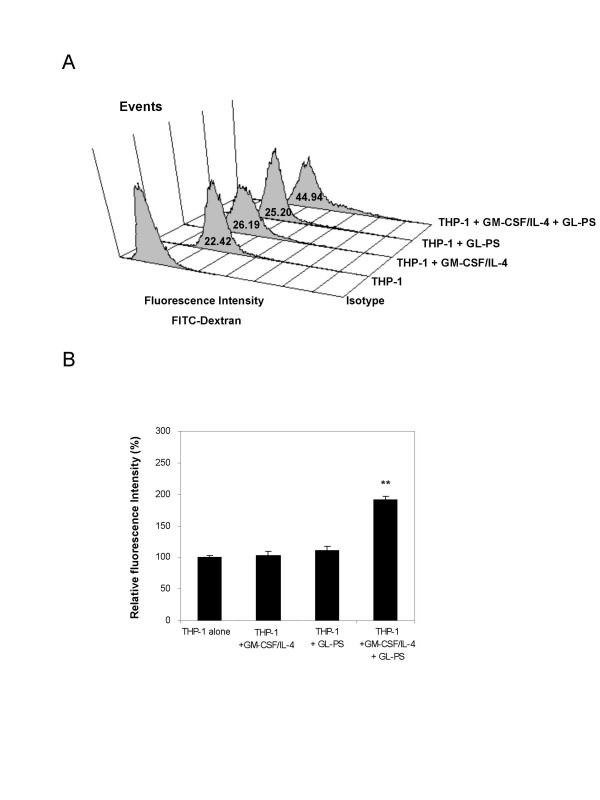
**Endocytosis of FITC-dextran by THP-1 cells and THP-1 DCs.** (**A**) Treated DCs were incubated with FITC-dextran for 1 h at 37°C and then washed for four times. Increase shift in fluorescence intensity (x-axis) was compared with the control, the one without FITC-dextran. The result was from one representative experiment of three independent repeats. (**B**) The increase in fluorescence intensity was calculated when compared with that of THP-1 cells alone after normalized with the background fluorescence, which was from the parallel experiments performed for all cells at 4°C. The results represented the mean ± SD from three independent experiments. ** *p *< 0.01 versus that of THP-1 cells.

We examined the endocytotic activity of the THP-1 DCs using fluorescent labeled FITC-dextran as antigens and incubated them at 37°C. We used PBS as negative control as well as parallel experiments at 4°C to serve as the background fluorescence. We found that the THP-1, THP-1 DCs with GM-CSF/IL-4 and THP-1 with GL-PS showed similar antigen uptake ability (Fig. 4A). For the GL-PS THP-1 DCs, we unexpectedly found that there was an increase in antigen uptake signals from the FITC-dextran after normalizing with the negative control (Fig. 4B). In order to rule out the possibility of upregulation of mannose receptor, which could account for the uptake of FITC-dextran, we determined the expression level of mannose-receptor and we found no change was observed (data not shown).

### Low IL-12 and IL-10 production in THP-1 DCs derived with GL-PS

**Figure 5:**
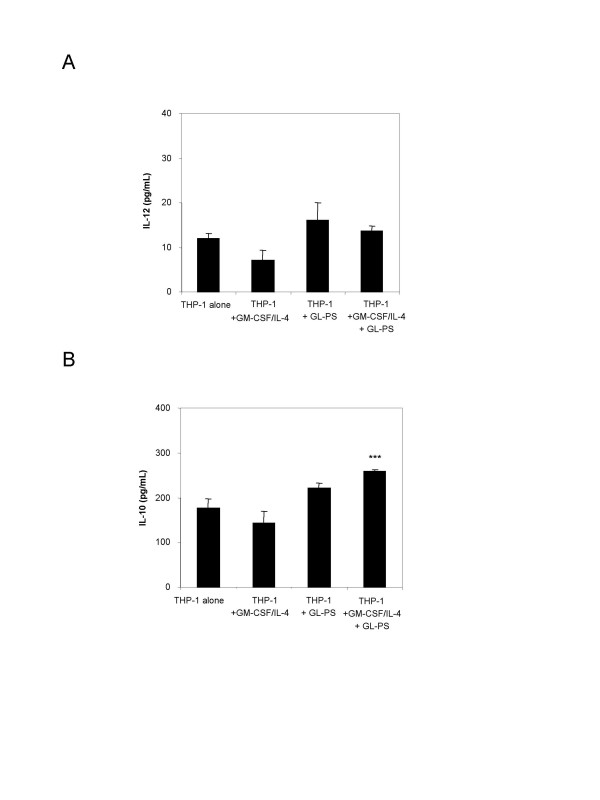
(**A**) **IL-12p70 and** (**B**) **IL-10 productions from THP-1 with and without GM-CSF/IL-4 or GL-PS**. The culture supernatants were collected and assayed by ELISA in duplicate. The detection ranges for IL-12 and IL-10 were 31.25–2000 pg/mL and 62.5–4000 pg/mL, respectively. The results represented the mean ± SD of four independent experiments for IL-12 and three independent experiments for IL-10. ***p < 0.001 versus that of THP-1 cells.

We detected low amount of IL-12 production from THP-1 cells, THP-1 DCs with GM-CSF/IL-4, THP-1 with GL-PS and GL-PS THP-1 DCs (Fig. 5A). Though THP-1 DCs with or without GL-PS had relatively higher IL-12 production, the amount was not significant. In contrast, GL-PS THP-1 DCs showed significant increase in IL-10 production (Fig. 5B).

### Decrease in T cell proliferation in allogeneic mixed lymphocyte reaction

**Figure 6:**
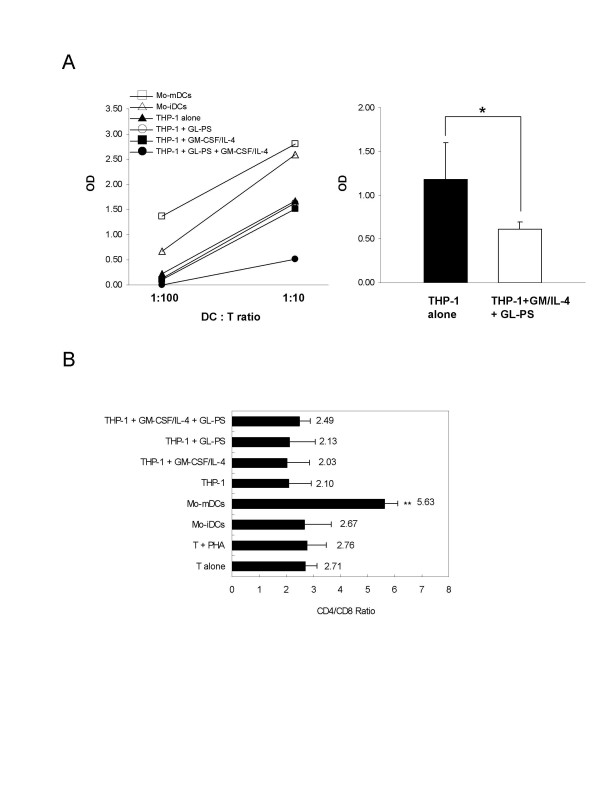
(**A) Allogeneic mixed lymphocyte  reaction of normal mature Mo-DCs (-c-); immature Mo-DCs (-r-); THP-1 cells  alone (-▲-); THP-1 cells with GL-PS (-○-); THP-1 DCs (-▪-) and GL-PS  THP-1 DCs (-●-) with CD3+ T cells in the ratio of 1:10 and 1:100**.****The optical densities of incorporated BrdU from DC:T co-cultures were normalized with that from T cells alone. The results represented one experimental result of three independent experiments. The results represented the mean ± SD of three independent experiments. *p < 0.05 versus that of THP-1 cells alone. (**B**) CD4/CD8 ratio of the co-cultured T cells. The co-cultured T cells so harvested were stained with CD4 and CD8 antibody and analyzed with flow cytometry. The results represented the mean ± SD of two independent experiments. **p < 0.01 versus that of T cells alone.

We determined the outcome of those THP-1 DCs when they were co-cultured with normal CD3^+ ^T cells, with immature and mature Mo-DCs as positive control. Significant increase in T cell proliferation was induced from immature to mature DCs (Fig. 6A, left). Interestingly, we found that there was a suppression of T cell proliferation in the co-culture of GL-PS THP-1 DCs when compared with other THP-1 cells or THP-1 DCs (Fig. 6A, left). When compared with THP-1 cells alone, the GL-PS THP-1 DCs showed significant decrease in T cell proliferation (Fig. 6A, right). We then examined that the suppression of T cell proliferation was not due to the induction of apoptosis (data not shown). The suppression did not focus on the CD4^+ ^helper T cells and CD8^+ ^cytotoxic T cells indicated by no significant change in CD4/CD8^+ ^ratio (Fig. 6B).

## Discussion

We demonstrated herein that purified immunomodulatory GL polysaccharides, which have been widely used as adjuvant therapy for anti-tumor purposes, could induce both monocytic leukemic cell proliferation and abnormal cellular differentiation in the form of immunoregulatory DCs. Interestingly, such proliferative stimulation was not found in other non-monocytic lymphoid and myeloid leukemic cell lines tested (data not shown), suggesting such effect was lineage specific. We also explored the possibility of using GL-PS to induce DCs from autologous blast cells in order to reduce leukemic cell burden.

We found that even among monocytic leukemic cells THP-1 and U937, there was a differential response to GL-PS. GL-PS only enhanced proliferation of U937, an AML M5 cell-line but could not induce its differentiation into DCs as in THP-1. Contrary to our findings, GL polysaccharides were reported to have to ability in inhibiting the growth of U937 cells, but it was under the influence of a conditioned medium primed by GL polysaccharides-stimulated human blood mononuclear cells [14]. This finding in fact suggested such inhibition required possible soluble factors secreted by the primed monocytes. In a recent report by Muller et al [15], GL was also demonstrated to be anti-proliferative in leukemic cells rather than inducing cell proliferation as shown in our study. This is mainly related to the choice of purified components being used. In our study, purified GL polysaccharides were used whereas purified GL triterpenes such as ganoderic acids were used in Muller et al. study. From the review of literature (see Table I), GL polysaccharides have consistently been shown to have immunological potency and can suppress cancer cell growth mainly by activating host's immune responses. In contrast, the triterpenes exert direct cytotoxic effect mainly through induction of cell cycle arrests and apoptosis in cancer cells including human leukemia, lymphoma and multiple myeloma (HL60, U937, K562, Blin-1, Nalm-6 and RPMI8226) [14,15]; breast cancer cells MDA-MB-231; and umbilical cord vascular endothelial cells HUVEC [16,17]. These data highlighted the importance of the choice of GL components selected for the study. Standardization of polysaccharides and triterpenes contents in the GL products has to be considered if we would like to extend these *in vitro* data into clinical study.

The GL-PS THP-1 DCs so generated morphologically resembled DCs with upregulated CD11c, HLA-DR, and costimulation molecules CD40, 80 and 86 (Fig. 2). Although the expression levels of these molecules were relatively low when compared with those on normal monocyte-derived DCs, they showed similar DCs function of stimulating allogeneic T cells proliferation responses. They were however immunoregulatory with the evidence of immature uptake of antigens, the IL-10 production as well as low potency in stimulating allogeneic T cell proliferation (Fig. 4, 5, 6). The suppressed T cell proliferation was believed related to their IL-10 production. IL-10 is an immunosuppressive cytokine and renders T cell stop proliferation even under the challenge of allogeneic differences [18]. This is also a mechanism for leukemic cells to escape from immune surveillance by dysregulation of immune systems via secretion of IL-10.

Previous studies demonstrated that GL polysaccharides could induce IL-1 release through the toll-like receptor (TLR)-4 signaling pathways in murine macrophages [19]. This raised a question that whether other TLRs ligands could account for the abnormal cellular responses on monocytic leukemic cells. To test this hypothesis, we also explored the effect of LPS (a ligand of TLR-4) and zymosan (a ligand of TLR-2 and dectin-1) on THP-1 DCs (data not shown). We found that LPS induced more significant cell adhesion to the culture plates and caused more cell death during cell harvesting. This phenomenon was also reported in previous studies but LPS could not induce the maturation of the leukemic DCs [8,9]. For the zymosan treated THP-1, there was no effect in expression of DC maturation markers; dextran-based endocytosis and IL-10, IL-12 productions. But we recognized that the zymosan treated THP-1 DCs with GM-CSF/IL-4 also had decrease potency in stimulating T cells proliferation. This implied that the leukemic cells THP-1 might respond to TLR ligands in different environments such as during infections and lead to abnormal changes.

## Conclusion

In summary, we found that GL polysaccharides could induce proliferation of monocytic leukemic cells. Together with GM-CSF/IL-4, GL-PS could induce THP-1 cells to become DCs with significant upregulation of antigen presentation and costimulation molecules expression. The immuno-potent nature was shown by the evidences that they retained ability to uptake antigens with IL-10 productions and decrease in immunostimualtory potential for T cell proliferation. Differential response of monocytic leukemic cells to GL-PS was observed. Our findings thus suggested that GL polysaccharides or other TLR ligands might have clinical impact on patients with monocytic leukemia. Whether GL-PS could induce DCs differentiation from autologous blast cells to help cancer patients to reduce cancer cell burden require further *in vivo *study for verification.

## Materials and methods

### Source and preparation of polysaccharides

Purified *Ganoderma lucidum *polysaccharides (GL-PS) was kindly provided by Prof. Lin ZB (Department of Pharmacology, Peking University Health Science Center, School of Basic Medical Sciences, Beijing, China). It is a polysaccharide peptide from GL mycelium with molecular weight of 584,900 and 17 amino acids. The ratio of polysaccharides to peptides is 93.51%: 6.49%. The polysaccharides consist of glucose, galactose, arabinose, xylose and mannose with molar ratios of 0.793:0.964:2.944:0.167:0.384:7.94 and linked by β-glycosidic linkages [20]. Endotoxin levels in GL-PS were constantly measured by using endotoxin-specific kinetic chromogenic Limulus Ameobyocyte Lysate (LAL) assay kit (Pyrochrome^®^, Associates of Cape Cod, Inc, East Falmouth, MA) with glucan inhibition buffer (Glucashield^®^, Associates of Cape Cod) to reconstitute the reagents according to the manufacturer's instructions. Standard curves were generated using Control Standard Endotoxin (CSE) and for better comparison, the LAL reactivity of β-glucan sample was also compared with that of lipopolysaccharide (LPS; Sigma). The endotoxin level of GL-PS was equivalent to 0.01% of 1 ng lipopolysaccharide, LPS, E. coli derived, suggesting negligible.

### Cell culture of leukemic cells

Leukemic cells, THP-1 and U937 were purchased from ATCC (Manassas, VA). It was characterized as AML M5. Cells were cultured in medium consisting of 90% RPMI 1640, 10% FBS, 100 U/mL penicillin, 100 mg/mL streptomycin (Invitrogen, Life Technologies, CA) and maintained at 37°C in a humidified atmosphere with 5% CO_2_.

### Generation of leukemic DCs *in vitro*

The generation of leukemic DCs was modified from that for normal Mo-DCs as previously described [11,21]. Leukemic cells THP-1 at the density of 1 × 10^5 ^per well were cultured with/without GL-PS (100 μg/mL) in the presence of GM-CSF (40 ng/mL; Novartis Pharma A6, Basle, Switzerland) and IL-4 (40 ng/mL; R&D Systems Inc, Minneapolis, MN) at 37°C under 5% CO_2_. On Day 3, 90% of the medium was replaced with fresh medium and cytokines. THP-1 DCs were then harvested on Day 5 and washed for further assays. For normal monocyte-derived DCs, mononuclear cells were isolated from buffy coat of healthy adult donors (Red Cross, Hong Kong SAR, China) by Ficoll-Paque Plus density gradient (Amersham Biosciences, Uppsala, Sweden). Monocytes were then isolated from PBMCs by positive selection using anti-CD14-conjugated magnetic microbeads (Miltenyi Biotec, Bergisch Gladbach, Germany). The isolated cells were cultured at a density of 1 × 10^6 ^cell/mL in RPMI 1640 medium supplemented with 10% FBS, 50 IU/mL penicillin and 50 IU/mL streptomycin (Invitrogen) with GM-CSF and IL-4 at 37°C under 5% CO_2 _for five days. CD3^+ ^T cells were isolated with the same method except using anti-CD3-conjugated magnetic microbeads (Miltenyi Biotec.). The purity of isolated monocytes was consistently > 85% while that of T cells was consistently > 98% as determined by Coulter Epics Flow Cytometer (Coulter Corporation, Miami, FL). Based on flow cytometry analysis, the immature DCs on Day 5 were 98.3% CD11c^+^CD1a^+ ^and 99.8% lineage negative (CD3^-^, CD14^-^, CD16^-^, CD19^-^, CD20^-^, CD56^-^).

### Cell proliferation assay

The effects of GL-PS on cell proliferation were measured using the Cell Proliferation Kit II XTT assay kit (Roche Molecular Biochemicals, Mannheim, Germany) according to the manufacturer's instructions. Briefly, 5 × 10^4 ^cells per well were grown in flat-bottom 96-well plates in a final volume of 100 μL culture medium overnight. Cells were then exposed to GL-PS at different concentrations (1 μg/mL to 1 mg/mL) for 24, 48 and 72 h. After the fixed time of incubation, 50 μL of the XTT labeling mixture was added to each well, and incubated for 4 h at 37°C in a humidified atmosphere with 5% CO_2_. The formation of formazan dyes in XTT labeling mixture by metabolically active cells was detected spectrophotometrically at 450 nm. The cell proliferation was calculated from the OD and expressed as percentage of negative control. To confirm the cell proliferation by increase in cell number, trypan blue exclusion assay was performed with trypan blue stain (Invitrogen). A minimum of 300 cells were counted under hemocytometer.

### Cell cycle analysis

GL-PS-treated leukemic cells were harvested, washed with PBS, fixed with ice-cold 70% ethanol and stored at 4°C. When for assay, the cell suspensions were incubated with RNase A (100 μg/mL; Sigma) and propidium iodide (4 μg/mL; Sigma) in PBS. Cell cycle phases were then analyzed with Coulter Epics Flow Cytometer (Beckman Coulter, Inc., Fullerton, CA). The percentage of G1, S and G2/M were determined with Cylchred Version 1.0.2 (Cardiff University, Wales, UK). For the expression of proliferating cell nuclear antigen (PCNA), we stained the cells with Fluorescein isothiocyanate (FITC) conjugated PCNA antibody (BD PharMingen, San Diego, CA) together with isotype control FITC-IgGκ (BD PharMingen). The cell were analyzed with the flow cytometer and the data were analyzed with WINMDI version 2.8 flow cytometry analysis software (Purdue University, West Lafayette, IN).

### Flow cytometry analysis of DCs

On Day 5, DCs were harvested, washed and labeled with fluorochrome-conjugated antibodies. After labeling, the cell suspension was washed and resuspended in 300 μL of 1% paraformaldehyde for flow cytometry. Fluorescein isothiocyanate (FITC), Phycoerythrin (PE) and Phycoerthrin-cyanin 5.1 (PC5)-conjugated isotype controls and CD14-PE, CD40-FITC, CD80-FITC, CD86-FITC, CD11c-PE and HLA-DR-PC5 antibodies were purchased from BD PharMingen. Flow cytometric analysis was performed with Coulter Epics Flow cytometer (Beckman Coulter) and analyzed with WINMDI software (Purdue University). To reduce inter-experimental variation, the mean fluorescence intensities for different CD markers were normalized with that of RPMI treated negative control as relative fluorescence intensity.

### FITC-dextran endocytosis assay

Leukemic cell-derived and monocyte-derived DCs were harvested and resuspended in RPMI with 10% FBS. FITC-dextran (molecular weight 40 kDa; Sigma) was added at a final concentration of 1 mg/mL. Cells were then incubated at 37°C or 4°C for 1 h. Thereafter, the cells were washed four times with cold PBS and then analyzed with flow cytometer.

### ELISA assay for cytokines

The supernatants from DCs cultures were collected after harvesting the cells and stored at -80°C until assayed for cytokines. The levels of IL-12p70 and IL-10 were then measured in duplicate with human Duoset^® ^ELISA Kit (R&D Systems Inc.). The detection ranges for IL-12 and IL-10 were 31.25–2000 pg/mL and 62.5–4000 pg/mL, respectively.

### Allogeneic mixed lymphocyte reaction

The leukemic cell-derived and monocyte-derived DCs were irradiated with a gamma-irradiator (Gammacell 1000 Elite, MDS Nordion Inc., Canada) at 30 Gy and co-cultured at the ratio of 1:10 with 1 × 10^5 ^allogeneic responder CD3^+^T cells in flat-bottom 96-well microtiter plates. Bromodeoxyuridine (BrdU) was added into the wells 16 h before the end of five-day culture. Cell proliferation during the last 16 h of the five-day culture was quantified by the Cell Proliferation ELISA, BrdU (colorimetric) kit (Roche Molecular Biochemicals).

### Statistical analysis

Comparisons between means were based on nonparametric Student's t test (2-tailed). For more than two groups, we compared the means with one-way ANOVA. The difference was statistically significant when *p *< 0.05.

## Competing interests

The authors declare that they have no competing interests.

## Authors' contributions

WKC designed, performed experiments and wrote up the manuscript; CCC performed experiments, KWL designed the experiments and revised manuscript, YLL provided materials and revised manuscript; GCFC designed, analyzed the data and wrote up the manuscript.

## References

[B1] Copeland DR, Silberberg Y, Pfefferbaum B (1983). Attitudes and practices of families of children in treatment for cancer. A cross-cultural study. Am J Pediatr Hematol Oncol.

[B2] Kelly KM, Jacobson JS, Kennedy DD, Braudt SM, Mallick M, Weiner MA (2000). Use of unconventional therapies by children with cancer at an urban medical center. J Pediatr Hematol Oncol.

[B3] Sparber A, Wootton JC (2001). Surveys of complementary and alternative medicine: Part II. Use of alternative and complementary cancer therapies. J Altern Complement Med.

[B4] Ramsay NA, Kenny MW, Davies G, Patel JP (2005). Complimentary and alternative medicine use among patients starting warfarin. Br J Haematol.

[B5] Chan G, Mullen P, Ha S, Wong G, Lee T, YL L (1998). Use of alternative medical treatments in paediatric oncology patients in Hong Kong. Annual Scientific Meeting of the Paediactric Society of Hong Kong.

[B6] Lin ZB, Zhang HN (2004). Anti-tumor and immunoregulatory activities of Ganoderma lucidum and its possible mechanisms. Acta Pharmacol Sin.

[B7] Wasser SP, Weis AL (1999). Therapeutic effects of substances occurring in higher Basidiomycetes mushrooms: a modern perspective. Crit Rev Immunol.

[B8] Berges C, Naujokat C, Tinapp S, Wieczorek H, Hoh A, Sadeghi M, Opelz G, Daniel V (2005). A cell line model for the differentiation of human dendritic cells. Biochem Biophys Res Commun.

[B9] Kim KD, Choi SC, Noh YW, Kim JW, Paik SG, Yang Y, Kim KI, Lim JS (2006). Impaired responses of leukemic dendritic cells derived from a human myeloid cell line to LPS stimulation. Exp Mol Med.

[B10] Cao LZ, Lin ZB (2002). Regulation on maturation and function of dendritic cells by Ganoderma lucidum polysaccharides. Immunology letters.

[B11] Chan WK, Lam DT, Law HK, Wong WT, Koo MW, Lau AS, Lau YL, Chan GC (2005). Ganoderma lucidum mycelium and spore extracts as natural adjuvants for immunotherapy. J Altern Complement Med.

[B12] Lin YL, Liang YC, Lee SS, Chiang BL (2005). Polysaccharide purified from Ganoderma lucidum induced activation and maturation of human monocyte-derived dendritic cells by the NF-kappaB and p38 mitogen-activated protein kinase pathways. Journal of leukocyte biology.

[B13] Masterson AJ, Sombroek CC, De Gruijl TD, Graus YM, Vliet HJ van der, Lougheed SM, Eertwegh AJ van den, Pinedo HM, Scheper RJ (2002). MUTZ-3, a human cell line model for the cytokine-induced differentiation of dendritic cells from CD34+ precursors. Blood.

[B14] Lieu CW, Lee SS, Wang SY (1992). The effect of Ganoderma lucidum on induction of differentiation in leukemic U937 cells. Anticancer Res.

[B15] Muller CI, Kumagai T, O'Kelly J, Seeram NP, Heber D, Koeffler HP (2006). Ganoderma lucidum causes apoptosis in leukemia, lymphoma and multiple myeloma cells. Leuk Res.

[B16] Lu QY, Sartippour MR, Brooks MN, Zhang Q, Hardy M, Go VL, Li FP, Heber D (2004). Ganoderma lucidum spore extract inhibits endothelial and breast cancer cells in vitro. Oncol Rep.

[B17] Sliva D, Sedlak M, Slivova V, Valachovicova T, Lloyd FP, Ho NW (2003). Biologic activity of spores and dried powder from Ganoderma lucidum for the inhibition of highly invasive human breast and prostate cancer cells. J Altern Complement Med.

[B18] Buggins AG, Milojkovic D, Arno MJ, Lea NC, Mufti GJ, Thomas NS, Hirst WJ (2001). Microenvironment produced by acute myeloid leukemia cells prevents T cell activation and proliferation by inhibition of NF-kappaB, c-Myc, and pRb pathways. J Immunol.

[B19] Hsu HY, Hua KF, Lin CC, Lin CH, Hsu J, Wong CH (2004). Extract of Reishi polysaccharides induces cytokine expression via TLR4-modulated protein kinase signaling pathways. J Immunol.

[B20] Shao BM, Dai H, Xu W, Lin ZB, Gao XM (2004). Immune receptors for polysaccharides from Ganoderma lucidum. Biochem Biophys Res Commun.

[B21] Liu E, Law HK, Lau YL (2003). BCG promotes cord blood monocyte-derived dendritic cell maturation with nuclear Rel-B up-regulation and cytosolic I kappa B alpha and beta degradation. Pediatric research.

